# Supplementary material for: From sequence to enzyme mechanism using multi-label machine learning
Source: BMC Bioinformatics. 2014 May 19;15:150. doi: 10.1186/1471-2105-15-150 (PMC4229970; doi:10.1186/1471-2105-15-150)
Supplement: Additional file 2 — Java code of ml2db. Additional file ml2db_code.tar.gz contains the Java source code to run the multi-label machine learning experiments and save the results to database. The code’s Javadoc is included. [file 1471-2105-15-150-S2.zip › additional file 2/ml2db/ml2db/doc/mulan/classifier/meta/thresholding/Meta.html]

Meta


JavaScript is disabled on your browser.


- Overview
- Package
- Class
- Use
- Tree
- Deprecated
- Index
- Help

- Prev Class
- Next Class

- Frames
- No Frames

- All Classes

- Summary:
- Nested |
- Field |
- Constr |
- Method

- Detail:
- Field |
- Constr |
- Method


mulan.classifier.meta.thresholding

## Class Meta

- java.lang.Object
- - mulan.classifier.MultiLabelLearnerBase
  - - mulan.classifier.meta.MultiLabelMetaLearner
    - - mulan.classifier.meta.thresholding.Meta

- All Implemented Interfaces:
  :   java.io.Serializable, MultiLabelLearner, weka.core.TechnicalInformationHandler

  Direct Known Subclasses:
  :   MetaLabeler, ThresholdPrediction

  ---

    

  ```
  public abstract class Meta
  extends MultiLabelMetaLearner
  ```

  Base class for instance-based prediction of a bipartition from the labels'
  scores

  Version:
  :   2010.12.14

  Author:
  :   Marios Ioannou, George Sakkas, Grigorios Tsoumakas

  See Also:
  :   Serialized Form

- - ### Constructor Summary

    Constructors

    Constructor and Description

    `Meta(MultiLabelLearner baseLearner,
    weka.classifiers.Classifier aClassifier,
    java.lang.String aMetaDatasetChoice)`

    Constructor that initializes the learner
  - ### Method Summary

    Methods

    | Modifier and Type | Method and Description |
    | --- | --- |
    | `weka.classifiers.Classifier` | `getClassifier()` Returns the classifier used to predict the number of labels/threshold |

    - ### Methods inherited from class mulan.classifier.meta.MultiLabelMetaLearner

      `getBaseLearner`
    - ### Methods inherited from class mulan.classifier.MultiLabelLearnerBase

      `build, getDebug, getTechnicalInformation, globalInfo, isUpdatable, makeCopy, makePrediction, setDebug`
    - ### Methods inherited from class java.lang.Object

      `equals, getClass, hashCode, notify, notifyAll, toString, wait, wait, wait`

- - ### Constructor Detail


    - #### Meta

      ```
      public Meta(MultiLabelLearner baseLearner,
          weka.classifiers.Classifier aClassifier,
          java.lang.String aMetaDatasetChoice)
      ```

      Constructor that initializes the learner

      Parameters:
      :   `baseLearner` - the MultiLabelLearner
      :   `aClassifier` - the learner that will predict the number of relevant
          labels or a threshold
      :   `aMetaDatasetChoice` - what features to use for predicting the number
          of relevant labels or a threshold
  - ### Method Detail


    - #### getClassifier

      ```
      public weka.classifiers.Classifier getClassifier()
      ```

      Returns the classifier used to predict the number of labels/threshold

      Returns:
      :   the classifier used to predict the number of labels/threshold


- Overview
- Package
- Class
- Use
- Tree
- Deprecated
- Index
- Help

- Prev Class
- Next Class

- Frames
- No Frames

- All Classes

- Summary:
- Nested |
- Field |
- Constr |
- Method

- Detail:
- Field |
- Constr |
- Method
